# Neo-Geometric Copper Nanocrystals by Competitive, Dual Surfactant-Mediated Facet Adsorption Controlling Skin Permeation

**DOI:** 10.3390/ma9120966

**Published:** 2016-11-28

**Authors:** Karmani Murugan, Yahya E. Choonara, Pradeep Kumar, Lisa C. du Toit, Viness Pillay

**Affiliations:** Wits Advanced Drug Delivery Platform Research Unit, Department of Pharmacy and Pharmacology, School of Therapeutic Sciences, Faculty of Health Sciences, University of the Witwatersrand, Johannesburg, 7 York Road, Parktown 2193, South Africa; karmani.murugan@students.wits.ac.za (K.M.); yahya.choonara@wits.ac.za (Y.E.C.); pradeep.kumar@wits.ac.za (P.K.); lisa.dutoit@wits.ac.za (L.C.d.T.)

**Keywords:** nanocrystals, nanoparticles, copper, geometric structure, self-assembly, transdermal, drug delivery

## Abstract

Neogeometric copper nanoparticles (CuNPs) have various applications yet its synthesis still proves to be challenging with regards to self-assembly and uniformity control. This study aimed to synthesize shape-specific CuNPs in the biomedical application of ascertaining skin permeation and retention of the CuNPs as a drug delivery system. The approach to the shape design involved the dual control of two surfactants to direct the shape organisation of the nanoparticles (NPs) while an interesting aspect of the study showed the competitive adsorption of the surfactants onto the nanocrystal facets to direct facet growth. The resulting copper nanoparticles were characterised using X-ray diffraction (XRD) and electron diffraction spectra analysis (EDS) for elemental and crystalline analysis. Thermogravimetric Analysis (TGA) identified the degradation of the surfactant coat and the synthesis of a novel copper-polymer complex and extensive transmission electron microscopy (TEM) was conducted to determine the nanoparticle morphology. Epidermal skin tissue served as the model for permeation studies of five idealistic nano-geometries and investigated its application in drug delivery with regards to cellular internalisation and transbarrier transport of the geometric CuNPs. A mechanistic consideration for shape control is discussed.

## 1. Introduction

Copper nanoparticles (CuNPs) have gained considerable research interest in the recent years for investigation in the biomedical and pharmaceutical sciences. The versatility of CuNPs facilitates its biomedical applications as a cytotoxic agent via oxidative stress [1,2], an antibacterial agent [3], an in vivo agent for imaging [4] as well as for advanced drug delivery applications [5]. In the last decade, researchers have proven that by manipulating the physicochemical, physicomechanical and morphological properties of CuNPs its industrial application expands and its extent of use. In particular, the geometrical shape and size of CuNPs has gained much interest [6]. Variation in the shape of CuNPs can be controlled via modification of synthesis processes and methods such as microemulsion [7], reverse micelle method [8], chemical reduction [9], electrochemical reduction [10], metal vaporisation [11], sonochemical processing [12], solution plasma methods [13] and microwave-assisted synthesis [14].

However, utilizing these methods does not ensure specificity in controlling the shape of CuNPs. In the bottom-up synthesis of shape controlled inorganic nanocrystals, studies have proven that colloidal solutions and key parameters such as temperature fluctuation, concentration of polymers and stabilizers, reducing agents, copper salt type and concentration, inert or ambient conditions, reaction time and water content are efficient for the minimal organisation of particle shape. The self-assembly control process is able to maintain the nano-shape within the colloidal dispersion by minimal chemical, physical or mechanical modification during synthesis. Despite these advances, it is still highly complex to optimise the shape of CuNPs.

A few studies have confirmed the novel synthesis parameters of metal nanoparticles (NPs) through the formation of shape-controlled NP fabrication. For instance, Sadjadi and co-workers [15] investigated the effect of temperature and reaction time on the morphology and aspect ratios of silver nano-rods. Pileni and co-workers [16] demonstrated the effect of water volume on the morphology of CuNPs. In order of low to high water content, spherical and cylindrical nanoparticles and planar-type lamellar were formed. Another group of researchers, Nomura and co-workers [8], varied the raw ion concentration and addition of ethanol in the synthesis of self-assembled barium chromate nano-wires, nanodots and nanorods. Spherical CuNPs and nanorods were achieved by De and Mandal [17] when altering the surfactant concentration.

Despite the significant studies on metallic NP shape, these have been limited to two or three self-assemblies per formulation synthesis, partial synthesis of microparticles or formulations not displaying complete shape homogeneity due to the high chemical reactivity of the surface of the copper metal. Therefore, this article focuses on the exploits of the surfactants to partially reduce and cap CuNPs to stabilize and simultaneously dictate NP shape to produce samples of homogenous geometries. The study aimed to synthesize CuNPs of distinct shapes by manipulating two surfactant concentrations with homogeneity throughout each colloidal solution. This research also reports on the successful thermo-chemical reduction in the organised synthesis of neogeometrical CuNPs and epidermal skin permeation kinetics based on nano-geometry.

## 2. Results and Discussion

CuNP synthesis involves the reduction of a copper precursor, Cu(II) species, by ascorbic acid in solution leading to the nucleation and growth of metal nanoparticles. In addition to its use as a reducing agent, ascorbic acid also functions as a capping agent [18,19].

### 2.1. Influence of Variation in Surfactant Concentration on Copper Nanoparticles (CuNPs) Shape

The scope for change and sensitivity to an external parameter was ascertained to determine the geometries that could be derived from the crystalline cubic structure of nanocrystals and to assist with the synthesis of uniform neogeometric CuNPs. After the nucleation of the Cu crystal, the growth of the nanocrystal facets is controlled to synthesize the various shapes. In this study, the major influence involved in the dictation of NP shape is the organisation of the surfactant molecules on specific faces to preferentially grow facets of certain dimensions and the induction of truncation of the CuNPs. The surfactants adsorb on the solid-liquid interface of particular nanocrystal facets imparting various energies on the different facets and the controlled, elevated reaction temperature allow for the deviation from the shape formation during synthesis using standard reaction parameters [20,21]. Previous research studies show that cetyltrimethylammonium bromide (CTAB) can act as a soft template, this study proves that the simultaneous use of various concentrations of CTAB and sodium dodecyl sulfate (SDS) in the presence of heat can dictate and control the shape of seeded copper NPs. The varied surfactant molar concentrations used to synthesize neogeometrical CuNPs are listed in Table 1.

**Figure 1:**
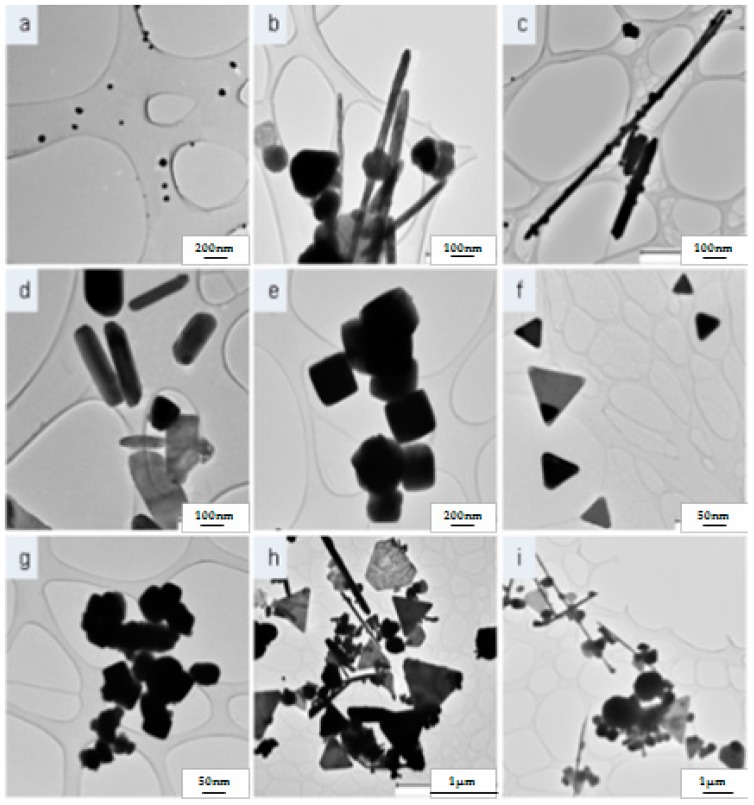

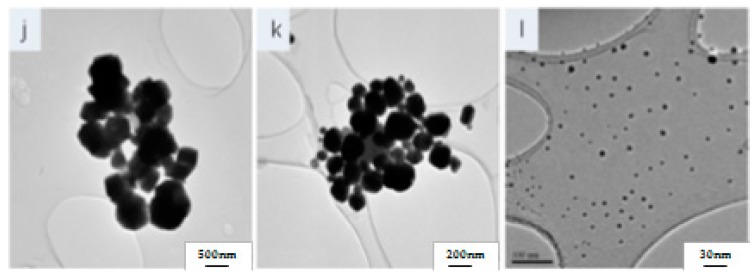
(**a**) Sample S1, Spherical nanoparticles (90 nm); (**b**) Sample S2, Combination of spheres and rods; (**c**) Sample S3, Combination spheres and rods with predominant rods; (**d**) Sample S4, Rod-like nanoparticles; (**e**) Sample S5, Cubic-shaped nanoparticles; (**f**) Sample S6, Pyramidal nanoparticles; (**g**) Sample S7, Irregular spherical particles; (**h**) Sample S8, Combination of neogeometrical nanoparticles; (**i**) Sample S9, Combination of neogeometrical nanoparticles at reduced size range; (**j**) Sample S10, Irregular nanospheres; (**k**) Sample S11, Spherical nanoparticles (250–300 nm); and (**l**) Sample S12, Spherical nanoparticles (10 nm).

As the Cu nanocrystals nucleate in aqueous solution, surfactant molecules adsorb onto the surface of facets dictating the shape formation by inhibiting or stimulating facet growth. It is suggested that shape of an face centred cubic (FCC) nanocrystal can be influenced by the ratio of the growth rates along the (100) and (111) directions [22,23]. According to Personick and co-workers [24], CTAB is a cationic surfactant which displays preferential facet adsorption altering growth rates along crystallographic planes. In water, the critical micelle concentration (CMC) of CTAB is 9 × 10^−4^ mmol/L and SDS is 8 × 10^−3^ M, rendering the number of surfactant molecules sufficient to provide a micellar environment for the newly synthesized CuNPs forming an adsorption layer around the NPs [25]. The crystallinity of the CuNPs is affirmed by high-resolution transmission electron microscopy (HR-TEM) (Figure S1), X-ray diffraction (XRD) (Figure S2) and electron diffraction spectra (EDS) analysis found in the Supplementary Materials. Table 1 and Figure 1 particularly demonstrate the dependence of surfactant concentration on NP shape formation. The relative percentage co-existence of the shapes in the poly disperse samples are obtained from a population of 100 NPs as per Anova analysis.

**Figure 2:**
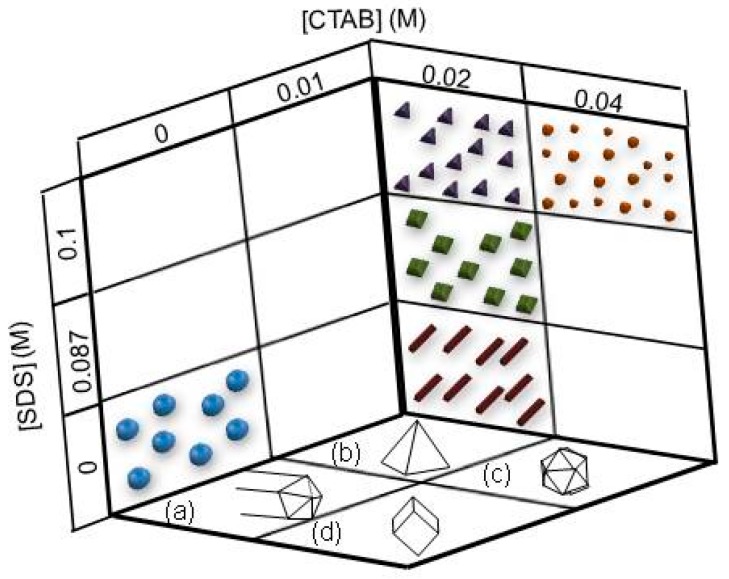
Schematic of growth of homogenous shapes according to surfactant variation: (**a**) Growth of a rod structure from the decahedral precursor; (**b**) tetrahedral precursor geometry; (**c**) polyhedral precursor geometry; and (**d**) cuboctahedral precursor geometry growing from the (100) and (111) planes. CTAB: cetyltrimethylammonium bromide; SDS: sodium dodecyl sulphate.

Whilst maintaining a constant concentration of CTAB, the difference in morphology using 0.02 M CTAB is far more sensitive than the samples using 0.01 M CTAB as shown in Figure 2. Homogenous samples of geometries with varying aspects from rods to cubes and pyramids can be attributed to the variation in SDS concentration. Upon addition of SDS whilst maintaining 0.02 M CTAB, the aspect ratio of the NPs showed a fundamental decrease in aspect ratio of the nano-rods and nano-cubes. Further increasing the SDS concentration reduced the number of geometric aspects resulting in a change from a four-aspect structure to a three-aspect structure and additionally reducing the size dimensions of the NPs. The number of sharp aspects increases and decreases spontaneously, showing responsiveness to increasing SDS in the presence of 0.02 M CTAB.

The morphology difference between different CuNP samples in the current study is a result of the interaction achieved by the coordination bonds between Cu ions, CTAB and SDS. Prior to nucleation of Cu, the CuSO_4_·5H_2_O salt interacts with CTAB and SDS molecules at concentrations above the critical micelle concentration (CMC) which then surrounds the Cu ions. The chemically adsorbed surfactant molecules and ions mediate the morphological changes of the CuNPs as well as act as stabilising agents by coating the nanocrystals and prevent oxidation of the metal Cu under ambient conditions [26]. The adsorption of surfactant promotes facet growth and, as shown in Table 1, facet growth is dependent on surfactant concentration. Considering this concept, copper nuclei are most stable in the cuboctahedral, decahedral and tetrahedral and polyhedral precursor nuclei and assume these geometries during synthesis [27].

The structure of the rod-shaped NP grows from a decahedron and exists with a five-fold symmetry [27]. The induction of the decahedron formation is mediated by the truncation of the five subunit edges of the decahedra nucleus in the (111) planes and additional intermediate faces in the (100) planes as shown in Figure 2a. Growth along the facet of the {100} surface results in an elongated structure with two 5-fold symmetry points on either end. The stable tetrahedral geometry with a threefold axis acts as the precursor nucleus in the formation of the pyramidal-shaped NPs [27] as shown in Figure 2b. The facets are dominated by {111} suggesting that the growth of the tetrahedral geometry is directed by this facet unlike the decahedron and cuboctahedron geometries. It can be assumed that the spherical NPs are formed from the truncated polyhedral precursor nuclei and are composed of several {100} and {111} facets as shown in Figure 2c. The cuboctahedral precursor is defined by its {100} and {111} facets as shown in Figure 2d. Cubic NP geometry arises from the selective growth of the {111} facet of the cuboctahedral nucleus. The selective growth of this facet suggests that it has a particularly higher surface energy than the {100} facet. Selective adsorption of surfactant on {100} retards its growth, allowing for the exaggerated growth of this slow growing surface resulting in a cubic geometry [27]. Slow growth of (100) results in this dominant facet producing cube shaped NPs.

In the absence of CTAB and SDS, spherical NPs are formed. It can be assumed that polyhedrons (Figure 2c) as the initial stable precursor nuclei with equal dimensions are formed. Thereafter, upon the addition of surfactant, there is preferential facet growth due to surface energy and surface adsorption. It is evident from other research [28] and these results that the surface energy varies between the different facets and displays competitive and selective adsorption of ions and molecules. The molecular and ionic affinity to certain facets could also be used to rationalise the geometry of nanocrystals. Due to the presence of the surfactants in the solution before, during and after the seeding process, the precursor nuclei geometries are uniform and can be controlled. After the seeding of the nuclei, the formation of the precursor nuclei is dictated by the type of surfactant molecules and ions and its concentration. Thereafter, its growth is also mediated by surface adsorption onto specific facets. In the study conducted by Pileni and co-workers [16], the research team suggested the reverse micelle synthesis of shaped copper NPs involves the initial formation of decahedral, cuboctohedral or tetrahedral precursor shape followed by the favoured adsorption of surfactants on the facets of the nanocyrstals to stimulate facet growth of certain dimensions. Thus, the growth rates of specific NP facets can be controlled during synthesis to direct the required nano-shape.

The {111} planes are more energetically favourable when directing tetrahedral growth compared to the decahedron and cuboctahedron which have high-energy facets specific to each geometry [23]. The results indicate that directing tetrahedral, decahedral or cuboctahedral growth can be achieved by the ionic and molecular adsorption on (100), (110) and (111) planes while spherical CuNPs are directed by polyhedral structures. The affinity of these ions and molecules to certain planes plays the key role in geometry dictation. Surfactant adsorption on a facet reduces the growth of the facet and the remaining facets grow steadily becoming the least dominant facet [22].

During the synthesis of blank copper nanocrystals (Sample S1), polyhedral precursor nuclei are directed. Excess ascorbic acid and its degradative products promote the growth of this crystal nucleus. When CTAB at 0.01 M and SDS at 0.087 M are introduced into solution, decahedral and polyhedral precursor nuclei are grown. This is a clear indication that competitive inhibition of facet adsorption occurs by CTAB and SDS. When CTAB is increased to 0.03 M at SDS 0.087 M, a sample consisting of combinatorial geometries are synthesized. It can be assumed that the increased CTAB reduces the growth of (111) to promote the tetrahedral structure, as well as the polyhedral structure. According to the results, when CTAB is used at 0.02 M, the geometry of the nanocrystals are the most responsive to surfactant concentration, hence, highly controlled. When CTAB at 0.01 M is reacted with SDS at maximum concentration (0.1 M), rod and spherical nanocrystals are formed. When compared to the sample synthesis of SDS at 0.087 M (42% rods, 58% spheres), the composition of rods and spheres are now significantly different (78% rods, 22% spheres) implying that the excess SDS competes for adsorption on the {100} facet when used simultaneously with CTAB since the rod:spherical nanocrystal ratio has increased.

Regarding the neogeometrical CuNP synthesis, it is suggested that at 0.02 M the aqueous surfactant environment is ideal for the generation of single-shaped geometrical precursor ions (Figure 2). Specific to 0.02 M CTAB, monodisperse rods, cubes and pyramids were formed in solution when compared to samples of other CTAB concentrations. The rod-shaped NPs indicate CTAB adsorption on the (100) plane which can be accurately presumed due to CTAB being the independent surfactant during nano-rod synthesis. Adsorption on (100) prolongs the growth of the facet allowing for the elongation of the decahedron as shown in Figure S1(1a). In the absence of SDS, the formation of the decahedral precursor structure is clearly dominant. Upon introducing 0.087 M SDS to 0.02 M CTAB the cuboctahedral precursor nuclei is directed and the effect of selective CTAB adsorption on (100) is maintained resulting in cube shaped nanocrystals. As shown by the results, when SDS is used at 0.1 M there is competitive inhibition of CTAB adsorbing onto the (100) facet. The SDS at 0.1 M when used simultaneously with 0.02 M CTAB promotes the synthesis of the tetrahedral precursor stimulating the synthesis of pyramidal nanocrystals, adsorbing on the (111) facet.

When CTAB is used at 0.03 M in the absence of SDS, the formation of irregular polyhedrons is evident as shown in Figure 1j. This is shows the non-selective surfactant adsorption in this concentration range. Similar to the Sample S10 where CTAB is used independently at 0.04 M, the higher concentration can be rewarded of its excess by producing more regular surface adsorption and also reducing the crystal size by 100 nm. CTAB at 0.03 M and SDS at 0.087 and 0.1 M both produce samples various geometries showing non-selective precursor nuclei and facet growth. The increased SDS, however, is able to control the size of all geometries indefinitely. The tetrahedral size reduction is 400 nm, rods by an aspect ratio of 7 and spheres by 50 nm in diameter.

At the specific CTAB concentration of 0.04 M, the growth of polyhedrons is constant. The excess CTAB adsorbs on all faces unselectively resulting in a geometrical structure with several facets. The addition of SDS at 0.087 M partially assists with the control of CTAB adsorption equally on facets resulting in polyhedral nanostructures that are slightly more regular than spheres. At 0.04 M CTAB, 0.087 M SDS also affects the size of the nanocrystal, reducing the polyhedron diameter by 200 nm affirming the use of SDS as a size controlling agent in the synthesis of nanocrystals [17]. When SDS is used in an excess of 0.1 M at CTAB 0.04 M, the polyhedral structure is at its most stable spherical structure. The SDS enhances equal adsorption of CTAB while reducing the nanocrystal size significantly by 90 nm.

### 2.2. Surface Charge Analysis

Zeta potential, as an indicator of surface charge, gives the probability of stability of the sample. The greater the potential of the CuNPs, the higher the stability and reduced likeliness to aggregate due to the repel of the charged NPs. Ascorbic acid has a negative charge, hence Sample S1 has a negative potential [29]. The reducing agent also serves as a stabilising agent by binding to the surface of the synthesized nanocrystal, preventing aggregation of the NPs [20]. The results indicate a change in surface charge with different surfactant types and concentrations. The use of cationic CTAB and anionic SDS manipulates the surface charge from the standard negative charge (Sample S1 = −28.3 mV) of the CuNPs synthesized without surfactant as shown in Table 2. The surface charge of the CuNPs synthesized with CTAB as the independent surfactant (S4, S7 and S10) are fairly stable with positive potentials and the least stable values are due to the high concentrations of both surfactants simultaneously used in CuNP synthesis. Samples S2, S6, S8 and S11 where SDS is used at 0.087 or 0.1 M has characteristics of least stabile profiles and may possess the highest aggregation while relatively low concentrations of CTAB (S3, S5 and S9) displayed strongly negative charges as expected. The samples synthesized with one surfactant with a dominant concentration prove to be most stable with potentials less than −25 mV or exceeding +25 mV.

### 2.3. Yield Analysis

Yield analysis was conducted on the CuNPs samples with differing results as shown in Table 1. An additional aspect of interest noted in this study regarded the variation in yield between the samples. The percentage of CuNPs synthesized is influenced by the surfactants and possibly potentiates the synthesis of copper nanocrystals and enhances the capping effect. Typically, CTAB has been shown to increase the number of nanocrystals formed during reduction while using an independent reducing agent [30]. This confirms the use of CTAB as a catalyst reducing agent. CTAB samples prepared without ascorbic acid did not produce CuNPs reiterating that CTAB is not an independent reducing agent; however, samples that contained higher CTAB increased the CuNP yield. CuNP samples that contained a constant CTAB concentration varied in yield when the SDS concentration differed. The results show that SDS may have also functioned as a catalyst and the samples increased in yield with an increase in SDS concentration. Additionally, an increase in yield seen with SDS may be due to the surfactant adsorption on the crystal facets and coating of the NP surface.

### 2.4. Evaluation of Chemical and Structural Changes of the Surfactant-Coated Copper Nanoparticles

**Figure 3:**
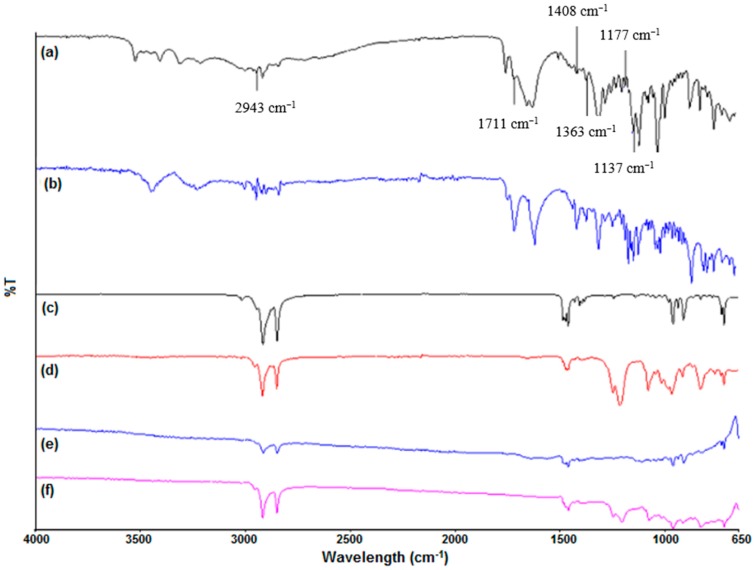
Fourier-Transform Infrared (FTIR) spectra of: (**a**) ascorbic acid; (**b**) Sample S1; (**c**) CTAB; (**d**) SDS; (**e**) Sample S4; and (**f**) Sample S5.

Fourier-Transform Infrared (FTIR) analysis was conducted on the reducing agent, surfactants and coated CuNPs to determine the adsorption of the excess ascorbic acid, CTAB and SDS on the CuNPs (Figure 3). The capping effect of the surfactants also promotes stability towards aggregation allowing for the optimal function of the NPS. Similar characteristic peaks found between ascorbic acid (Figure 3a) and Sample S1 (Figure 3b) and between CTAB (Figure 3c), SDS (Figure 3d), Sample S4 (Figure 3e) and S5 (Figure 3f) give an indication of the adsorption on the CuNPs. Common peaks found on the ascorbic acid and Sample S1 spectra include CH stretching at 2943 cm^−1^, carbonyl groups at 1711 cm^−1^, aromatic rings at 1408 cm^−1^, ether groups at 1137 and 1177 cm^−1^. The FTIR spectrum of Sample S4 (Figure 3e) and selective peaks of Sample S5 (Figure 3f) can be attributed to the adsorption of CTAB (Figure 3c) on the surface of the CuNPs. Both spectra exhibited CTAB characteristic peaks of CH bending in the 1400–1500 cm^−1^ region, stretching vibrations of C–CH2 in the methylene chains at 2848 cm^−1^ [31] and 2916 cm^−1^ [32]. Sample S5 displays a peak at 3018 cm^−1^ assigned to the antisymmetric stretching modes of the trimethylammonium headgroup of CTAB [32] indicating facet adsorption of CTAB. When comparing the spectrum of Sample S5 (Figure 3f) with the SDS (Figure 3d) spectra, several corresponding peaks are definite indicating the presence of the surfactant in the CuNP sample. The bands on spectrum Figure 3f correspond to the OSO_3_^−^ bands at 1215 and 1247 cm^−1^ [33] and peaks at 2918 and 2954 cm^−1^ assigned to the CH_2_ group on the SDS spectrum [34]. The FTIR data are indicative of reducing agent and surfactant adsorption on the CuNPs and is further corroborated by the following Thermogravimetric Analysis (TGA) data.

### 2.5. Thermal Degradation Analysis of Surfactant-Coated Copper Nanoparticles

**Figure 4:**
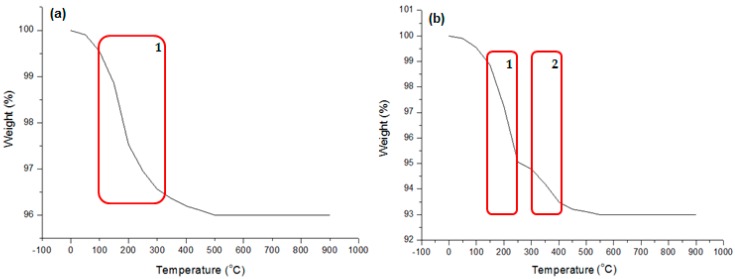
Thermogravimetric Analysis (TGA) curves of: (**a**) Sample S1 showing degradation of reducing agent (highlighted area in red); and (**b**) Sample S2 showing degradation of surfactants on the NP surface (highlighted area in red).

TGA was used to assess the relative composition of the capping agents on the CuNPs. Figure 4a,b shows the representative TGA curves obtained for Samples S1 and S2 which were heated from 30 to 900 °C in the presence of nitrogen gas. Figure 4a curve shows one key degradation point between 110 and 320 °C, which can be related to the decomposition of the ascorbic acid on the surface of the CuNPs. Sample S2 (Figure 4b), capped with ascorbic acid, CTAB and SDS, displays two key degradation steps. The first weight loss step in Figure 4b curve occurs between 110 and 240 °C indicating the degradation of ascorbic acid as the capping agent and between 300 and 410 °C, which is attributed to the degradation of the adsorbed surfactants. All remaining samples have TGA profiles similar to Figure 4b.

The degradation of the ascorbic acid in Sample S1 occurs at temperatures between 100 and 320 °C and between 110 and 240 °C in Sample S2. These data indicate the capping effect of the ascorbic acid and the intramolecular interactions between ascorbic acid and the surfactants resulting in a slightly raised degradation temperature in Sample S2. After analysis at the end temperature of 900 °C, an average of 95.7% of sample remains. Copper degrades at temperatures higher than 900 °C indicating that the degradation that occurred is attributed to the supporting surfactants in the samples and that the synthesized CuNPs are thermally stable. The change in mass can be translated to the ratio of capping agents to CuNPs. A shell of about 2% ascorbic acid and 6% surfactant adsorption was determined from thermogravimetric analysis, resulting in 93%–96.4% of the mass due to the Cu.

### 2.6. Effect of Nano-Shape on Skin Permeation through Excised Mice Skin

**Figure 5:**
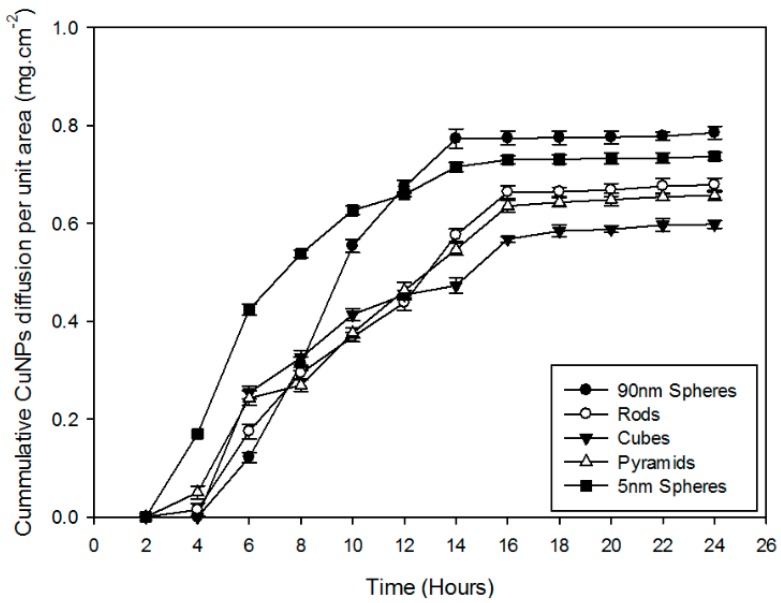
Ex vivo permeation profiles of geometric copper nanoparticles through excised BALB/c mice dermal tissue (*n* = 3).

Permeation studies were conducted to determine the effect of nanoparticle geometry on transcellular drug delivery. Pre- and post-ex vivo characterisation, conductivity analyses confirmed maintenance of skin integrity of the mice skin tissue samples. Analyses of the ex vivo permeation results (Figure 5) within the first 2 h show the CuNPs reaching a state of equilibrium in the epidermal tissue. No CuNPs permeation is detected in the first hour and a mere 0.0149 mg·cm^−2^ CuNPs is detected in the rod NPs sample, 0.05 mg·cm^−2^ of the pyramid NPs sample and 0.17 mg·cm^−2^ of the 5 nm sphere NP sample at 2 h. The first CuNP detection of the 100 nm spheres and cubes occurs at 4 h at 0.122 and 0.254 mg·cm^−2^, respectively. The permeation lag can be explained by the CuNPs reaching a state of concentration equilibrium in the epidermal tissue and thereafter permeating into the receptor compartment. Interestingly, the skin permeation and transcellular transport due to shape kinetics of the spheres are higher than the other geometrical NPs. The 90 nm spheres have the highest cumulative diffusion per unit area (0.78 mg·cm^−2^) (Figure 5) but have a lower NP flux (4.20 × 10^−2^), as shown in Table 3, compared to the 5 nm spheres which have a lower cumulative diffusion per unit area (0.73 mg·cm^−2^) with a higher NP flux (5.88 × 10^−2^). The initial CuNP permeation of the 5 nm spheres supersedes the 90 nm spheres and eventually stabilizes at a concentration below the 90 nm spheres while the rods have an initial lower flux than the pyramids and cubes but steadily increase with all geometries reaching system equilibrium after 14–16 h. The 5 nm spheres show a long burst release permeation as opposed to the rods, pyramids and cubes which is followed by the 90 nm spheres. Interestingly, the pyramids show a short burst release until 6 h, thereafter reaching a stable flux.

Figure 5 confirms the hypothesis of the nano-shape effects on cellular permeation where the geometrical structure of the NP affects cellular and transdermal permeation. Cellular internalisation and epidermal tissue localisation of the CuNPs also show nano-shape to be a dictating factor when considering this drug delivery system for specific transdermal or dermal use. Table 3 lists the concentration of neogeometric CuNPs retained by the epidermal tissue post-internalisation after analysis using inductively coupled plasma optical emission spectrometry (ICP-OES). The cube-shaped CuNPs shows the highest retained concentration at 0.34 mg with 90 nm spheres at 0.09 mg. Having the highest retention potential, cubes also have the lowest permeation potential with an average flux indicating a system application for epidermal drug delivery. The 90 nm spheres retain the least amount of CuNPs but have the highest permeation with an above average flux when compared with other neogeometric CuNPs giving the probability of its use as a transdermal drug delivery system. The alternative nano-shapes can be dually used as systems requiring dermal and transdermal drug delivery depending on application specificities as can be extrapolated from Figure 5 and Table 3. The results of the CuNPs retained correlate with the CuNPs diffusion through the epidermal skin tissue. The high permeation of the CuNPs accounts for the low concentration of CuNPs retained and vice versa. The results do not indicate a superior system for drug delivery but rather results based application of the neogeometric CuNPs. Previous studies identified hollow copper-sulphide nanoparticles as a skin disruption mechanism to enhance transdermal drug delivery of human growth hormone upon application of a near-infrared laser [35]. In addition, Ahamed and associates [36] recently studied the antibacterial effects of CuNPs which can be included in medical devices similar to the study conducted by Sankar and co-workers [37] who investigated the antibacterial effects of CuNPs on wound healing. Having the benefit of inhibiting pathogenic bacterial growth, the future goals of this study will also focus on the toxic properties of CuNPs in transdermal drug delivery.

## 3. Materials and Methods

Copper II sulphate pentahydrate (CuSO_4_·5H_2_O) was purchased from Merck (Darmstadt, Germany); hexadecetyl trimethylammonium bromide (CTAB) and sodium dodecyl sulphate (SDS) were purchased from Sigma-Aldrich Co. (Aldrich, Steinheim, Germany); L-ascorbic acid (98%) was purchased from Roche (Johannesburg, South Africa); and trypsin/EDTA was purchased from Lonza (Walkersville, MD, USA). All water used for the synthesis was deionized. All chemicals were analytical grade and used without further purification.

### 3.1. Synthesis of Copper Nanostructures

The thermal-reduction method of synthesizing nanocrystals included a standard CuSO_4_·5H_2_O solution added to varied molar concentrations of aliquot hexadecetyl trimethylammonium bromide (CTAB) and sodium dodecyl sulphate (SDS) solutions heated at 50 °C. Solution was further heated between 85 and 90 °C and a standard ascorbic acid solution was added as the reducing agent in a drop-wise manner to allow for spontaneous copper formation without a reducing agent overload. The temperature was then maintained at 80 °C using a mercury thermometer (Brannon Thermometers, Cumbria, UK).

### 3.2. Determination of the Effect of Surfactant Concentration on Particle Morphology

TEM (FEI T12 Spirit Transmission Electron Microscope (120 kV), Hillsborough, CA, USA) and High-Resolution TEM (JEOL JEM 2100F (200 kV), Tokyo, Japan) confirmed the synthesis of self-assembled neogoemetrical NPs. Samples were also subjected to TEM-Energy Dispersive Spectroscopy (EDS) for high-speed elemental analysis.

### 3.3. Crystallinity and Composition Characterisation of Synthesized Cu

A powder X-ray diffractometer (XRD) (MiniFlex 600, Tokyo, Japan) was used to monitor diffraction patterns of the CuNP samples to validate the synthesis of crystalline copper. A continuous scan rate of 0.1°/min from 0° to 90° was used with Cu Kα radiation (λ = 1.54 Å).

### 3.4. Determination of the Stability of the CuNPs

The zeta potential measured the surface charge of the CuNPs, thus indicating the stability and aggregation potential of the NPs. Each suspension was diluted 1:15 in distilled water, filtered through a 0.22 μm Millipore filter, transferred into a capillary cell and was analysed by a Zeta sizer (DTS (nano), Malvern instruments Ltd., Worcestershire, UK).

### 3.5. Molecular and Structural Transitional Analysis of the Surfactant-Coated Copper Nanoparticles

Fourier-Transform Infrared (FTIR) analysis of ascorbic acid, CTAB, SDS and synthesized polymer-coated CuNPs was undertaken to evaluate and compare vibrational characteristics of the chemical functional groups in response to infrared light interactions. FTIR spectra were recorded on a Perkin Elmer Spectrum 2000 FTIR spectrometer with a MIRTGS detector (PerkinElmer Spectrum 100, Wales, UK) at a wavenumber range of 650–4000 cm^−1^ with a resolution of 4 cm^−1^ and 10 scans per spectrum.

### 3.6. Determination of Nanoparticle Yield

Yield studies were conducted to elucidate the effect of surfactants in the investigation of Cu reduction. The yield of the CuNPs was determined by weighing the dried CuNP samples using an electronic balance (Mettler, Model AE 240, Greifensee, Switzerland) with readings recorded to 2 decimal places. Percentage yield was calculated using Equations (1)–(3).

Number of moles = *C* × *V* (1)

Reacting mass of Cu (theoretical yield) = *n* × *M* (2)

(3) $$\%\;Yield=\;\frac{actual\;yield}{theoretical\;yield}\times 100$$

### 3.7. Thermal Degradation Analysis of the Surface Surfactant-Coating of the CuNPs

Thermogravimetric Analysis (TGA) using a 4000 TGA (PerkinElmer Inc., Waltham, MA, USA) was conducted to evaluate the polymer-coating of the CuNP samples as the function of temperature in nitrogen atmosphere under a flow of 40 mL/min and heating rate of 10 °C/min from 30 to 900 °C. The Pyris 6 software (PerkinElmer Inc., Waltham, MA, USA) was used to perform the thermal analysis. Each test consisted of a powder weighing approximately 10–20 mg and produced similar results among all samples.

### 3.8. Ex Vivo Permeation Evaluation of Five Distinct Nano-Shapes

Ex vivo studies were conducted to determine the skin permeation efficiency of five nanosystems with the most constructive structures. The study served to identify the nano-shape with the highest potential of crossing the stratum corneum barrier and entering the epidermal layer of BALB/c mice skin samples. The hair from the dorsal aspect of the skin was shaved and the skin was thereafter excised. The skin was washed and incubated in Trypsin/EDTA at 37 °C for 2 h in an incubator controlled in a 5% CO_2_ environment allowing for the detachment of the dermal layer thereafter. Skin integrity testing was conducted before and after permeation studies using a Seven Multi S40 pH/electrical conductivity meter (Mettler-Toledo, Zurich, Switzerland). The permeation studies were carried out utilizing a Franz Diffusion Cell (FDC) apparatus (PermeGear Inc., Bethlehem, PA, USA) equipped with a 12 mL receptor compartment, clamp, stirrer-bar and a thermostat controlled water jacket. Epidermal BALB/c mice skin samples were placed between the donor and receptor compartments of the FDC. Samples of simulated plasma (100 µL) in the receptor compartment (PBS; 12 mL; pH 7.4; 37 °C) were withdrawn at suitable time intervals over 24 h. Post permeation studies, the skins were digested with nitric acid at 70 °C for analysis to determine copper content retained by the dermal skin tissue. Receptor samples and skin samples were analysed by Inductively Coupled Plasma-Optical Emission Spectroscopy (Activa S) (Horiba Scientific, Munich, Germany).

### 3.9. Data Analysis and Confirmation of Statistical Significance of All Assays Performed

All synthesis and characterization studies including TEM analysis, nanoparticle counting, XRD, zeta potential, yield, TGA and ex vivo permeation studies were carried out in 3 independent experiments in duplicates for each evaluation. Data were expressed as mean (±standard error (SE)) and analyzed by 1-way analysis of variance (ANOVA). *p* values less than 0.05 was considered statistically significant.

## 4. Conclusions

The fabrication of non-agglomerated, monodispersed and exquisite geometrically organised CuNPs has been successfully demonstrated by reducing a copper salt using ascorbic acid in the presence of two key surfactants. Homogenous samples of cubes, pyramids, rods and spheres were synthesized proving shape control of the method. Particle morphologies could be controlled by manipulating the surfactant concentration, as well as by utilizing the degradation products of ascorbic acid to assist with capping and shape dictation of the NPs. It can be concluded that CTAB at 0.02 and 0.04 M are most stable when synthesizing homogenous geometries in one pot. The SDS variation thereafter plays the key role in inhibiting or stimulating the adsorption of CTAB by its own adsorption. The function of the SDS can be assumed to be a vital one as an additional geometry and size dictator. The shape organisation of the CuNPs is remarkable despite the small increase in surfactant concentration between the samples. The epidermal permeation, flux and tissue-retained CuNPs clearly show a shape-dominated trend and the drug delivery uses of the CuNPs thereof prove to be varied. This approach in the synthesis of neogeometric copper nanocrystals offers the controlled shape formation of copper nanocrystals and reproducibility of heterogeneous and homogenous shape-formation based on the surfactant concentration. In addition to nano-shape, the surface chemistry of the CuNPs also affects ex vivo kinetics related to drug delivery and will be considered for functionalisation. The CuNPs will be further applied in drug delivery as a function of its shape and toxicity. Considering the CuNPs retaining potential or transbarrier transport, the nanosystems may be applied to but not limited to skin conditions such as skin cancer.

## Figures and Tables

**Table 1:** Surfactant variations corresponding to physicochemical characteristics of synthesized CuNPs, Data presented are mean ± SE of three experiments performed in duplicate. * *p* ≤ 0.05.

Formulation	[CTAB] (M)	[SDS] (M)	Yield (%)	Geometrical Structures	Size of Nanostructures (nm)	Figure
S1 ^a^	0.00	0.000	81.0	Spheres	90 ± 9	2a
S2 ^b^	0.01	0.087	83.6	Combination	Shape dependent	2b
S3 ^b^	0.01	0.100	85.0	Spheres and rods	Shape dependent	2c
S4 ^a^	0.02	0.000	86.0	Rods	150 × 20	2d
S5 ^a^	0.02	0.087	89.8	Cubes	200 × 200 ± 13	2e
S6 ^a^	0.02	0.100	90.5	Pyramids	100 ± 12	2f
S7 ^b^	0.03	0.000	87.0	Irregular spheres	300 ± 11	2g
S8 ^b^	0.03	0.087	92.2	Combination	Shape dependent	2h
S9 ^b^	0.03	0.100	96.2	Combination	Shape dependent	2i
S10 ^b^	0.04	0.000	89.7	Irregular spheres	500 ± 8	2j
S11 ^b^	0.04	0.087	96.8	Irregular spheres	200–250 ± 10	2k
S12 ^a^	0.04	0.100	97.1	Spheres	5 ± 3	2l

^a^ Sample with ideal, homogenous nano-geometries; ^b^ Sample with irregular or combination geometries. CuNPs: copper nanoparticles; CTAB: cetyltrimethylammonium bromide; SE: standard error; SDS: sodium dodecyl sulphate.

**Table 2:** Zeta potential data collated from all CuNPs samples.

Formulation	[CTAB]	[SDS]	Zeta Potential (mV)
S1	0.00	0.000	−28.3
S2	0.01	0.087	−19.9
S3	0.01	0.100	−25.7
S4	0.02	0.000	26.9
S5	0.02	0.087	−30.3
S6	0.02	0.100	−21.3
S7	0.03	0.000	27.1
S8	0.03	0.087	−6.75
S9	0.03	0.100	−34.5
S10	0.04	0.000	33.1
S11	0.04	0.087	−18.4
S12	0.04	0.100	16.7

**Table 3:** Copper nanoparticle flux and associated permeability coefficients for the geometric copper nanoparticles through excised BALB/c mice dermal tissue (*n* = 3).

Formulation	Geometrical Structure	Flux, J_S_, (mg·cm^−2^·hr^−1^)	CuNPs Retained in Dermal Tissue (mg)
S1	Spheres (90 nm)	4.20 × 10^−2^	0.09
S4	Rods	3.61 × 10^−2^	0.14
S5	Cubes	3.75 × 10^−2^	0.34
S6	Pyramids	2.66 × 10^−2^	0.28
S12	Spheres (5 nm)	5.88 × 10^−2^	0.11

## Supplementary Materials

The following are available online at www.mdpi.com/1996-1944/9/12/966/s1. Figure S1: (1a) Rod-shaped nanoparticle and inset showing 5-fold center of the decahedral geometry at (111); (1b) lattice fringes and spacing of rod-shaped nanoparticle; (1c) uniform SAED patterns; (2a) Cube-shaped nanoparticle; (2b) lattice fringes and spacing of cube-shaped nanoparticle (2c) uniform SAED patterns; (3a) Pyramid-shaped nanoparticle; (3b) lattice fringes and spacing of pyramid-shaped nanoparticle; (3c) uniform SAED patterns; (4a) Spherically-shaped nanoparticles; (4b) lattice fringes and spacing of spherical nanoparticles; (4c) uniform SAED patterns, Figure S2: (a) Powder X-ray diffraction patterns of synthesized copper nanocrystals; (b) Energy Dispersive Spectra showing pure elemental copper.
