# Supplementary material for: Neo-Geometric Copper Nanocrystals by Competitive, Dual Surfactant-Mediated Facet Adsorption Controlling Skin Permeation
Source: Materials (Basel). 2016 Nov 28;9(12):966. doi: 10.3390/ma9120966 (PMC5456976; doi:10.3390/ma9120966)
Supplement: Supplementary file 1 [file materials-09-00966-s001.pdf]

Supplementary Materials: Neo-Geometric Copper Nanocrystals by Competitive, Dual Surfactant-Mediated Facet Adsorption Controlling Skin Permeation

Karmani Murugan, Yahya E. Choonara, Pradeep Kumar, Lisa C. du Toit and Viness Pillay

1. Nanocrystal Lattice Spacing

Individual NP geometry and crystallinity was analysed to determine the lattice fringe spacing. A single rod nanocrystal (Figure S1(1a)) consists of lattice fringes along (111) with a spacing of 2.09 Å and (100) with 1.81 Å as shown in Figure S1(1b) indicating the copper nanocrystals are of FCC structure. The inset in Figure S1(1a) clearly shows the 5-fold centre of the rod tip with the rod orientated to display its {111} facet. Figure S1(2a) shows a single cube-shaped nanocrystal consisting of an inter-fringe distance of 1.81 Å which is attributed to the lattice space along (100) (Figure S1(2b)). The pyramidal-shaped NP (Figure S1(3a)) shows a monodisperse 2.09 Å (111) lattice fringe (Figure S1(3b)). Spherical nanocrystals (Figure S1(4a)) have lattice fringes along (111) with a spacing of 2.09 Å and (100) with 1.81 Å as shown in Figure S1(4b). Selected area electron diffraction (SAED) patterns of the single nanocrystal illustrate ordered diffraction spots indicating high crystalline features of the capped CuNPs (Figure S1(1c, 2c, 3c, 4c)).

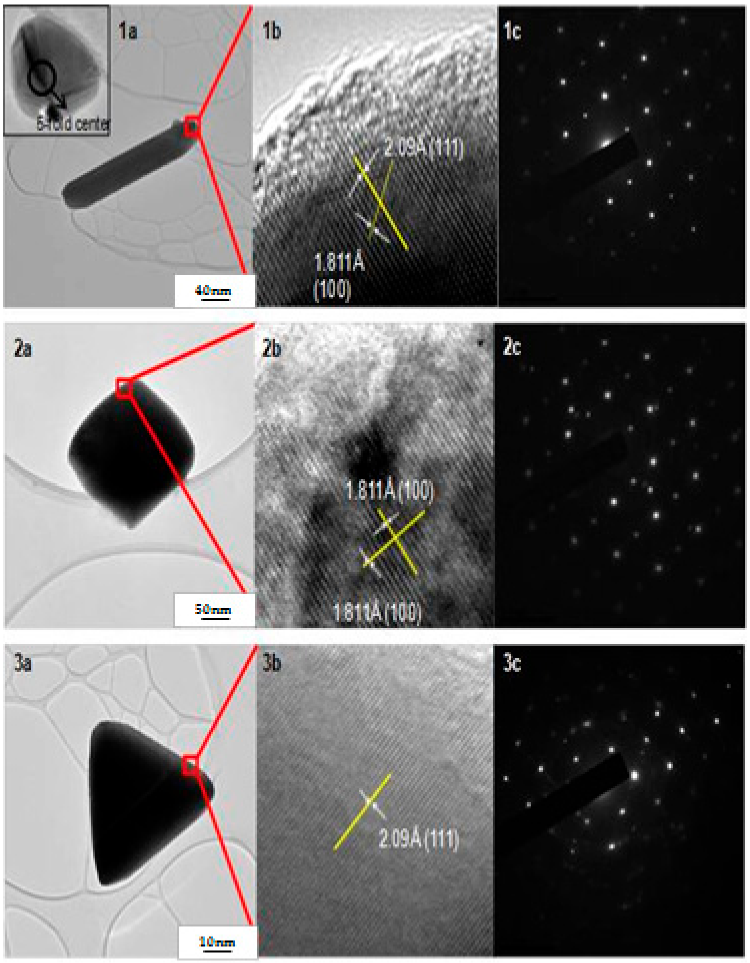

Figure S1. *Cont.*

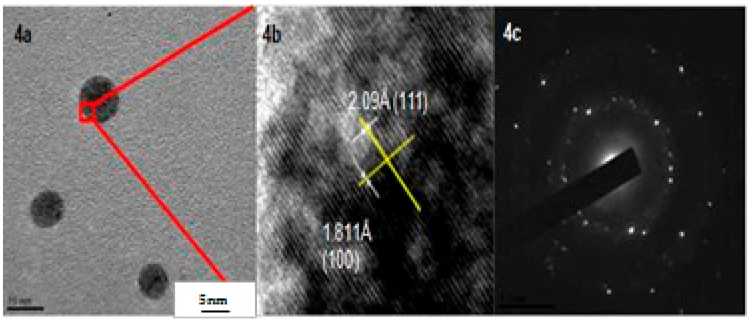

Figure S1. (**1a**) Rod-shaped nanoparticle and inset showing 5-fold center of the decahedral geometry at (111); (**1b**) lattice fringes and spacing of rod-shaped nanoparticle; (**1c**) uniform selected area electron diffraction (SAED) patterns; (**2a**) Cube-shaped nanoparticle; (**2b**) lattice fringes and spacing of cube-shaped nanoparticle (**2c**) uniform SAED patterns; (**3a**) Pyramid-shaped nanoparticle; (**3b**) lattice fringes and spacing of pyramid-shaped nanoparticle; (**3c**) uniform SAED patterns; (**4a**) Spherically-shaped nanoparticles; (**4b**) lattice fringes and spacing of spherical nanoparticles; (**4c**) uniform SAED patterns.

2. X-ray and Electron Diffraction Analysis

The powder X-ray diffraction patterns of the CuNPs as shown in Figure S2a are indicative of highly oriented crystalline CuNPs and are similar amongst all samples. It corresponds to phase-pure Cu according to the literature pattern (JCPDS, File No. 04-0836) with strong, prominent peaks at 43.1°, 50.3° and 74° correlating to (111), (200) and (220) planes of copper crystals, respectively. The 100% pure-crystalline Cu characteristic peaks are indexed to a face-centred cubic (FCC) crystal structure. A small proportion of impurity, copper oxide, was detected at 35.5° (002) and is owing to formation of copper oxide following interactions with air during the XRD analysis.

As indicated by enlarged views, rods (Figure S1(1a)), cubes (Figure S1(2a)), pyramids (Figure S1(3a)) and spheres (Figure S1(4a)) have well-defined edges and aspects. The lattice fringes and SAED patterns corresponding to Figure S1(1a–4c) indicate FCC copper and differ according to their individual orientation. According to the Bragg equation (Equation (1)),

$$n\lambda = 2d\sin(\theta) \quad (1)$$

can be rearranged to determine the spacing, d, between corresponding lattices (Equation (2)),

$$d = n \times \frac{wavelength}{2sin(\theta)} \quad (2)$$

Using θ from the crystalline peaks arising from the X-ray diffraction data (Figure S2a), the lattice distance can be calculated from the (111), (200), (220) and (311) planes as 2.09 Å (0.209 nm), 1.81 Å (0.181 nm), 1.27 Å (0.127 nm) and 1.09 Å (0.109 nm) respectively. These values correspond ideally with the figures associated to the lattice fringe spaces giving a clear indication that copper of the correct domains were formed.

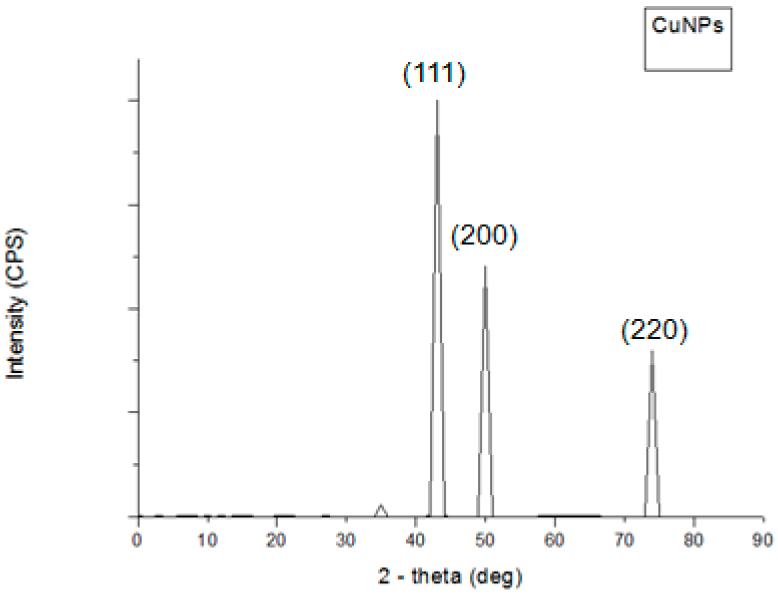

(**a**)

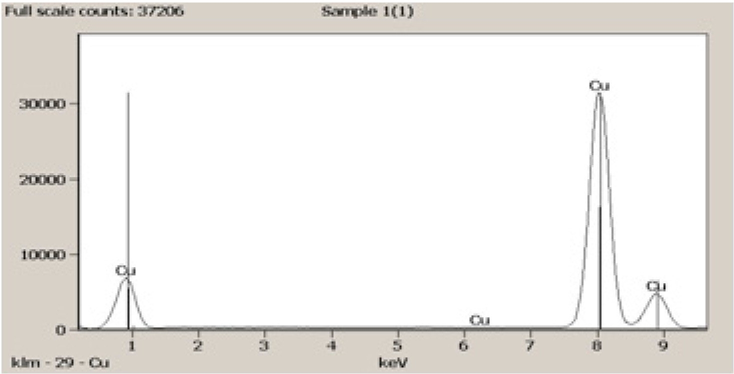

Element Line	Weight %	Weight % Error
Cu K	100.00	±0.40
Cu L	---	---
Total	100.00	

(**b**)

Figure S2. (**a**) Powder X-ray diffraction patterns of synthesized copper nanocrystals; (**b**) Energy Dispersive Spectra showing pure elemental copper.

The energy dispersive spectra plots of CuNPs (Figure S2b) exclusively exhibits the characteristic peaks of ideal elemental Cu. Strong signals of Cu atoms show at a dominant $K\alpha_1$ peak at 8.0477 keV followed by a smaller $K\beta_1$ peak 8.0905 keV. The peak at 0.9498 keV demonstrates the $L\beta_1$ peak for electrons in the L-shell. The EDS plots for all CuNPs samples resulted in similar spectra. Thus, both metal characterisation analysis indicate the synthesis of pure copper. The strong FCC copper peaks from XRD and elemental copper peaks EDS (Figure S2b) demonstrates that the thermal-chemical reduction method used in conjunction with ascorbic acid serving the dual function of reducing agent and capping agent and two surfactants results in the synthesis of pure copper nanocrystals without additional impure phases.
